# Waveform Similarity Analysis: A Simple Template Comparing Approach for Detecting and Quantifying Noisy Evoked Compound Action Potentials

**DOI:** 10.1371/journal.pone.0136992

**Published:** 2015-09-01

**Authors:** Jason Robert Potas, Newton Gonçalves de Castro, Ted Maddess, Marcio Nogueira de Souza

**Affiliations:** 1 Department of Neuroscience, John Curtin School of Medical Research, Australian National University, Canberra, ACT, Australia; 2 Medical School, Australian National University, Canberra, ACT, Australia; 3 Instituto de Ciências Biomédicas, Universidade Federal do Rio de Janeiro, Rio de Janeiro, RJ, Brazil; 4 Biomedical Engineering Program, COPPE, Federal University of Rio de Janeiro, Rio de Janeiro, RJ, Brazil; University of Amsterdam, NETHERLANDS

## Abstract

Experimental electrophysiological assessment of evoked responses from regenerating nerves is challenging due to the typical complex response of events dispersed over various latencies and poor signal-to-noise ratio. Our objective was to automate the detection of compound action potential events and derive their latencies and magnitudes using a simple cross-correlation template comparison approach. For this, we developed an algorithm called Waveform Similarity Analysis. To test the algorithm, challenging signals were generated *in vivo* by stimulating sural and sciatic nerves, whilst recording evoked potentials at the sciatic nerve and tibialis anterior muscle, respectively, in animals recovering from sciatic nerve transection. Our template for the algorithm was generated based on responses evoked from the intact side. We also simulated noisy signals and examined the output of the Waveform Similarity Analysis algorithm with imperfect templates. Signals were detected and quantified using Waveform Similarity Analysis, which was compared to event detection, latency and magnitude measurements of the same signals performed by a trained observer, a process we called Trained Eye Analysis. The Waveform Similarity Analysis algorithm could successfully detect and quantify simple or complex responses from nerve and muscle compound action potentials of intact or regenerated nerves. Incorrectly specifying the template outperformed Trained Eye Analysis for predicting signal amplitude, but produced consistent latency errors for the simulated signals examined. Compared to the trained eye, Waveform Similarity Analysis is automatic, objective, does not rely on the observer to identify and/or measure peaks, and can detect small clustered events even when signal-to-noise ratio is poor. Waveform Similarity Analysis provides a simple, reliable and convenient approach to quantify latencies and magnitudes of complex waveforms and therefore serves as a useful tool for studying evoked compound action potentials in neural regeneration studies.

## Introduction

Peripheral nerve injury can lead to severe sensory and motor functional deficits. Progress in developing new treatments heavily relies on the evaluation of nerve function, and thus an objective, reliable and sensitive measure of nerve functional integrity is essential to accurately assess treatment outcomes. During nerve regeneration that follows injury, the nerve compound action potential (CAP) is fragmented, resulting in a noisy and complex CAP response comprised of smaller multiple compound events with sporadic latencies [1,2]. The aetiology of these complex events evoked from a single stimulus in the regenerating nerve warrants investigation, as it may provide us with clues on how we can improve the regenerative process. Accurate and consistent quantification of these complex events is therefore essential for such investigations. Despite the reproducibility of events evoked from regenerating nerves, there is little consistency among responses evoked from the regenerating nerves of different animals of the same species, and often very little resemblance to responses evoked from an intact nerve, at least during the earlier stages of recovery [2]. Furthermore, amplitudes of these events are of the same order of magnitude as background noise levels, and this problem is classically overcome by averaging multiple evoked responses. However, it may be necessary to average up to 500 evoked responses in order to reveal the response from the background noise [2,3], and test protocols with such large numbers of repeated stimuli risk compromising the functional integrity of regenerating nerves.

Manually quantifying complex responses comprised of multiple individual events with magnitudes near background levels is laborious and requires decision making criteria to discriminate between signal and background noise. These decisions are often based on rigid thresholds with hard inclusion/exclusion cut-off criteria. In reality, a complex response contains a broad spectrum of individual events and defining criteria to identify them can become an arbitrary task. Studies to date have therefore had to limit their analyses to qualitative descriptive comparisons of the distribution of individual events within a complex response [2–5].

A common strategy for the electrophysiological assessment of the recovering peripheral nerve is to measure its peak-to-peak amplitude and latency [2,3,6–8]. When presented with complex responses, it is common to select a single event, such as the first positive event [2–6], and/or the largest event [4,9]. However, the choice of which event to quantify is arbitrary and necessarily excludes other events of the complex response that may contain valuable information. In order to more accurately assess the success of regeneration after nerve lesion, an objective, sensitive and reliable technique is therefore essential, enabling robust assessment of electrophysiological responses.

We therefore set out to provide an analytical tool that facilitates the rapid and accurate quantification of noisy signals that may contain multiple compound events using a template-matching approach. Template-matching approaches are commonly incorporated in more sophisticated algorithms for spike sorting individual neuronal action potentials from a population of events [10–12]. While useful for identifying and classifying events, these approaches are not designed to quantify response magnitudes. Here we present a simple, objective, reliable and sensitive automated algorithm, named Waveform Similarity Analysis (WSA), which can unambiguously detect and quantify compound events within a complex response in terms of latency and magnitudes. WSA works by locating and quantifying similarity between a template signal and a signal of interest. We generated signals with low and high signal-to-noise ratios and successfully demonstrated that the output of the WSA algorithm was comparable to measurements performed by a trained expert; referred to as Trained Eye Analysis (TEA). WSA is a convenient tool for evaluating simple and/or complex signals, such as for example, responses evoked from regenerating nervous and/or muscle tissues.

## Materials and Methods

### Experimental protocol (*in vivo*)

Electrophysiological data was obtained from 9 rats that underwent sciatic nerve injury and 12 weeks of recovery following experimental injury. This study was carried out in strict accordance with the recommendations in the Guide for the Care and Use of Laboratory Animals of the National Institutes of Health. The protocol was approved by the Australian National University Animal Experimentation Ethics Committee (protocol number: RBSB0710), and all efforts were made to minimize suffering. Rats were anesthetised with ketamine/xylazine (80/5 mg/kg i.p.) and the right sciatic nerve was dissected and a 4 mm segment of the sciatic nerve was removed. The proximal and distal stumps were held together by a latex bridge which maintained the 4 mm gap between the two stumps. The left sciatic nerve remained intact. Rats were returned to their home cage and housed separately during the post-operative care period when they received antibiotic treatment (Cephazolin, 25 mg/kg) twice daily for 7 days.

After 12 weeks recovery, rats were anesthetised with urethane (1.4 g/kg i.p.), and placed on a temperature controlled heating pad to maintain body temperature at 37°C. The sciatic nerve was exposed at the level of the gluteal muscle on both sides. CAPs from nerve (CNAPs) were obtained with the use of a single hook-shaped recording electrode placed proximal to the nerve transection located under the gluteal muscle. The reference electrode was placed into the adjacent tissues located medially to the recording electrode. The sural nerve, a nerve void of motor fibres, was isolated near the ankle joint, and a bipolar electrode was placed at that location for stimulation. This allowed exclusive and direct stimulation of sensory cutaneous afferents. Both the recording and stimulating electrodes were isolated from surrounding tissues with mineral oil at body temperature.

To demonstrate that WSA can consistently evaluate CAPs of a different morphology to that evoked from the sural nerve, we analysed muscle CAPs from the tibialis anterior (CMAP). This was achieved by stimulating the sciatic nerve with the bipolar hook stimulating electrode at the same location as the recording site described for the CNAP protocol above. The CMAP was obtained from an electrode placed in the centre of the tibialis anterior muscle belly, with the reference electrode located in the connective tissue of the knee joint.

### Stimulation protocols and data acquisition (*in vivo*)

All stimulating pulses were generated using a Digitimer D4030, delivered by an isolated stimulator (Digitimer) 5 ms after the onset of the recording sweep. Pulses were applied to the sural (to generate CNAPs) and sciatic (to generate CMAPs) nerves, and current confirmed by measuring voltage across a 28 Ohm resister in series with the nerves. To evoke nerve responses of different magnitudes, a 0.2 ms square pulse stimulus of varying amplitudes (0.1 mA, 0.2 mA, 0.4 mA, 0.5 mA, 0.7 mA, 1.1 mA and 1.4 mA) was applied directly onto the nerve. The average of 10 evoked CNAP responses (N10avR) was obtained using sweeps of 50 ms duration, and with a 2 s recovery period between each stimulus. The N10avR was recorded 3 times for each stimulus intensity. To evoke a consistent variation of CMAP amplitude, a conditioning pulse stimulus (0.7 mA and 0.2 ms) was applied directly onto the nerve which was followed by an identical test pulse at one of 8 intervals (0.3, 0.6, 1, 2, 3, 5, 7 or 10 ms). A 50 ms sweep duration was used to record the average of 5 evoked CMAP responses (M5avR), but this was acquired only once. We therefore generated 3 noisy N10avRs and a single N5avR for each stimulus paradigm which were used for subsequent analysis by WSA and TEA.

All CNAP and CMAP responses were collected using an analogue oscilloscope (Tektronix 5113, 5A26) to view, amplify and filter (low pass 10kHz) the signal, which was subsequently converted to a digital signal (12 bits, 40 kHz sampling frequency) and saved as.txt files for subsequent processing and analysis with MATLAB.

### Simple and complex CAP evoked responses

Here we define a simple response as a CAP waveform composed of one obvious mono-, bi- or tri-polar event. An example of a simple response would be a CAP derived from a normal intact nerve or muscle. A complex CAP response is defined as one that contains several events within the total response, all individual events being reproducible and occurring at the same latency when evoked by an identical stimulus. An example of a complex response would be that of a regenerating nerve, where a response to a single stimulus contains several individual events.

### Waveform Similarity Analysis to measure CAP responses

WSA is based on the cross-correlation between two signals, which is a measure of similarity between two signals as a function of the time-lag between them. For discrete signals *x(m)* and *y(m)*, the cross-correlation for a time-lag *n* is defined as:

$$xycorr(x,y,n)=\sum_{m=-\infty }^{\infty }x^{*}(m)y(n+m)$$

where x*(m) denotes the complex conjugate of *x*(*m*).

Consider two equal signals shifted in time, where one signal is stationary and other dislocates in time over the other, producing a varying time-lag *(n)* between them. The maximum (peak) cross-correlation occurs when the time-lag between the two signals is zero, and indicates the initial shift between the two signals. When the time-lag between the signals is zero, the positive peaks of signals *x(m)* and *y(m)* are aligned, and thus maximally contributing to *xycorr*. Similarly, when the negative peaks of signals *x(m)* and *y(m)* align, they also make a positive contribution to *xycorr*. Thus, the maximum of the cross-correlation function will occur when the two signals overlap, and will depend on the magnitude of the two signals, i.e., the peak-to-peak magnitude, or energy, of both signals.

### Generation of template signals (*in vivo*)

**Fig 1:**
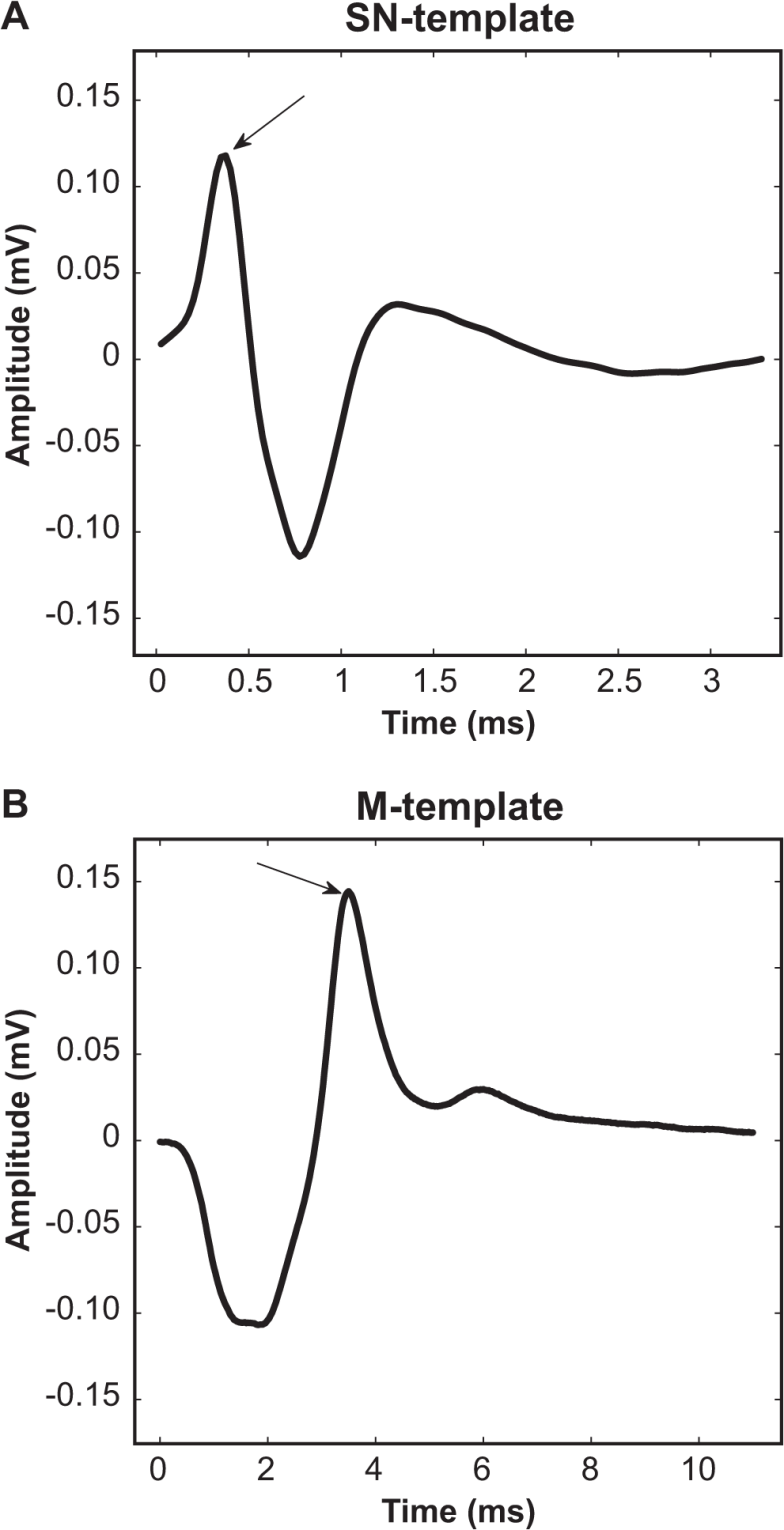
Generation of template signals used for waveform similarity analysis. Sural nerve (A) and tibial muscle EMG (B) template signals were generated from the intact side (left) of rats. The mean for each template was derived after individual responses were temporally aligned to the primary positive peak (arrows). See text for further details.

The sural nerve template signal (Fig 1A, SN-template) was obtained by averaging individual responses recorded from intact (left) sciatic nerves (mid-thigh level) evoked by stimulation (square pulse, 0.7 mA, 0.2 ms) of sural nerves from all animals subject to evaluation. The mean was derived after temporally aligning the primary positive peak (Fig 1A, arrow) of each individual response.

Similarly, the tibial muscle EMG template response (Fig 1B, M-template) was obtained by averaging individual tibial EMGs subsequent to temporal alignment of the positive peak (Fig 1B, arrow) evoked by stimulation (square pulse, 0.7 mA, 0.2 ms) of the intact (left) sciatic nerve of all animals subject to evaluation.

### Event detection and quantification derived by Waveform Similarity Analysis (*in vivo*)

Signals derived from the sural nerves and tibial muscles elicited from injured and intact sciatic nerves were used to test the WSA algorithm. The N10avR evoked from the injured nerve produced the noisiest signal for analysis, whilst the left M5avR produced the cleanest signal. All signals were DC shifted prior to analysis by subtracting the mean of the signal during the first 5 ms before the pulse stimulus from the entire signal.

**Fig 2:**
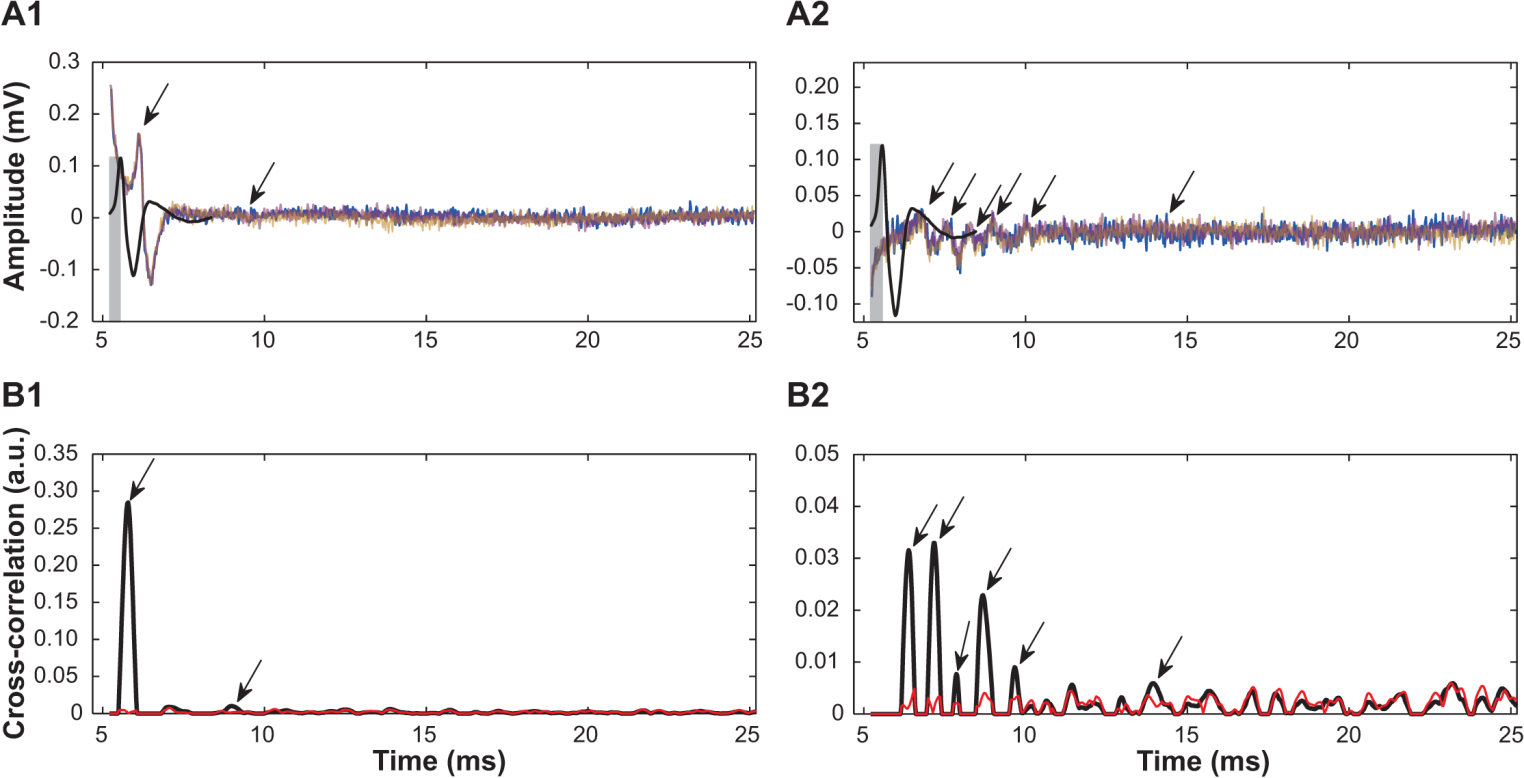
Example of event detection, latency and response magnitude of sural CNAPs derived by WSA. Row A illustrates three N10avRs (repeats are colour coded) to a 0.7 mA stimulus of an intact (A1) and regenerating (A2) nerve from the same animal, with the SN-template (see Fig 1A) superimposed (black). The cross-correlation was calculated for each instant between the SN-template and the N10avR, by sliding the SN-template trace across each N10avR. Row B illustrates the mean (black) and one standard deviation (red) of the cross-correlations calculated between SN-template and the three repeated N10avRs for an intact (B1) and a regenerating (B2) nerve of the same animal. An event was considered detected when the peaks of the mean of 3 cross-correlations was ≥ a detection threshold level (see Fig 3 for calculation of the detection threshold level). The latency for each event was given by the time of each respective mean cross-correlation peak (arrows row B) plus the 0.35 ms time lag between the onset and peak of SN-template (grey bars indicate latency correction, row A). The magnitude of each event was quantified as the peak of the mean cross-correlation for each event detected (arrows, B1 and B2), and is proportional to the energy of the event measured in arbitrary units (amplitude correction not shown). Total magnitude of each N10avR was calculated as the sum of the magnitudes of all events in N10avR (sum of all peaks indicated by arrows in B1 and B2).

**Fig 3:**
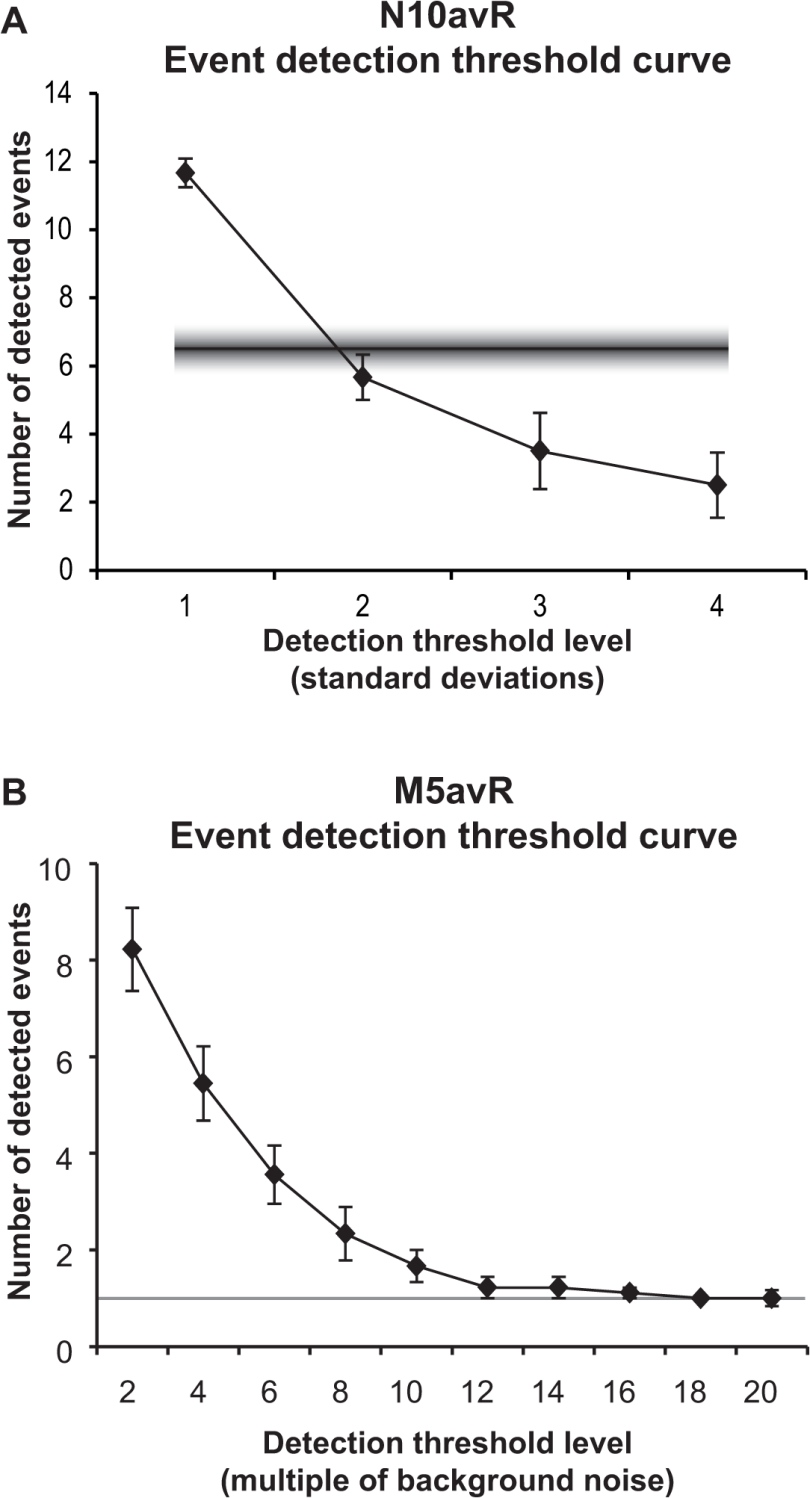
Effect of WSA event detection threshold levels on the number of identified events. Empirically derived functions were used to determine an ideal detection threshold. A demonstrates the mean number of events detected by WSA from N10avRs evoked from injured sciatic nerves by a 0.7 mA stimulus, as a function of the detection threshold level. The detection threshold level indicates the multiple of standard deviations (refer to red line in Fig 2B2 showing one standard deviation) that the mean (black line, Fig 2B2) of 3 repeated N10avRs had to be greater than, in order to detect an event using WSA. The fuzzy line represents the mean and SEM (6.5 ± 0.6) of the number of events detected by TEA for the same responses. Throughout the study, 2.0× was chosen as the standard detection threshold level for N10avRs, as this level detects a number of events not significantly different to that detected by TEA. B demonstrates the mean number of events detected from M5avRs evoked from injured sciatic nerves by a paired stimulus as a function of background noise level. The paired stimulus was 0.7 mA with an inter-pulse interval of 1 ms. Background noise was calculated from the mean absolute amplitude of the last 17 ms of the sweep, a region where no signal was present. The detection threshold level indicates the threshold based on a multiple of background noise. The grey line indicates the ideal level of event detection, i.e. where the number of events = 1. For *in vivo* experiments, 12× the background noise level was chosen as the standard detection threshold level for M5avRs.

The WSA algorithm determined the cross-correlation sequence between the response being quantified and its relevant template signal (Fig 1A or 1B). A plot containing cross-correlation peaks corresponding to the time instants of greatest similarity was produced by varying the time-lag between a nerve or muscle response and its template waveforms (Fig 2). As there were three N10avR and one M5avR generated per stimulus, the application of WSA to detect and quantify nerve and muscle CAPs were different. For nerve responses, this sequence was generated from the three N10avRs (Fig 2, row A) to produce a mean and standard deviation cross-correlation function (Fig 2, row B). Event detection and quantification for nerve responses were derived from cross-correlation function peaks where the mean was greater than 2 times the standard deviation. This detection threshold level was chosen as it detected a similar number of events as that detected by the trained eye (see Fig 3A).

The cross-correlation peaks correspond to the time lag at the beginning of the template. Latency correction (indicated by grey bars, Fig 2) was therefore applied by adding the latency difference between the template peak and template onset, to the latency of the peaks of the cross-correlation sequence. The magnitudes of the cross-correlation peaks are proportional to the energy of the signal. To enable direct comparisons with TEA, an amplitude correction constant was determined post-hoc by comparing the mean outputs from WSA and the mean values from TEA, and multiplying this value to the output of the WSA.

**Fig 4:**
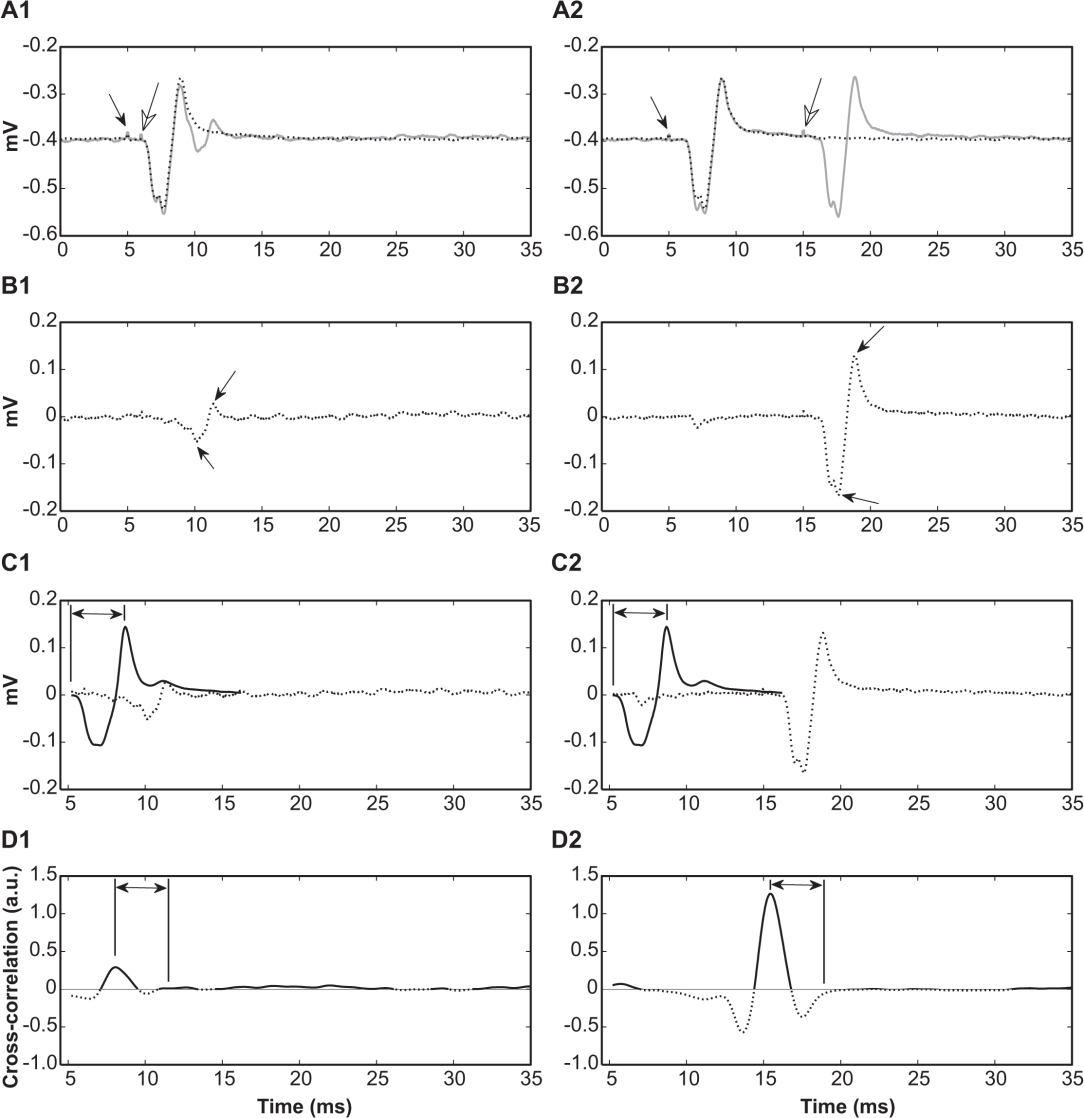
Pre-processing and quantification of CMAP responses by WSA and TEA. Shown are examples of paired conditioning (black arrow, row A) and test (white arrow, row A) stimuli presented to an intact sciatic nerve, with inter-pulse intervals of 1 ms (column 1) and 10 ms delays (column 2) of the same animal. First, an average of 2 repeated M5avRs were evoked by a single conditioning pulse stimulus (0.7 mA 0.2 ms) at 5.0 ms after the onset of the recording sweep (black arrow, row A) in the absence of a following test pulse. This average trace (dotted traces, row A) was subsequently subtracted from each M5avR evoked by the paired stimuli (grey traces, row A) to reveal the response to the test pulse in the absence of the conditioning pulse (row B), and subsequently quantified by TEA (peak-to-peak, arrows, row B) and WSA methods (rows C and D). The template comparing wave, M-template (black trace, row C) was slid across the pre-processed M5avR (dotted trace, row C) to derive the cross-correlation function between the M-template and the pre-processed M5avR signals at each time instant (row D, positive cross-correlations black, negative cross-correlations dotted). Positive peak values of the cross-correlation sequence represent arbitrary units (a.u.) of the event magnitude (amplitude correction not shown), and the latency is measured as the time at the cross-correlation peak plus the 3.5 ms time lag (latency correction, indicated by double arrows, row D) between the onset and peak of M-template (double arrows, rows C).

For muscle responses, only a single M5avR was generated for each paired pulse stimulus. To examine the M5avR evoked by the test pulse without interference from the conditioning pulse, it was necessary to subtract the response evoked by the conditioning pulse (at 5.0 ms after sweep onset) from the response evoked by the paired stimulation (conditioning + test) pulse (Fig **4**). The response to the conditioning pulse alone was obtained by averaging 2 M5avRs prior to the commencement of all stimulation protocols. WSA was therefore performed on a single pre-processed M5avR waveform. The threshold detection level was derived from an empirically derived function of background noise levels (Fig 3B), and latency and amplitude correction also applied as described for N10avRs above.

The cross-correlation functions served 3 purposes: (1) the peaks above thresholds served to identify events. (2) The latency of the respective event could be calculated as the time to the cross-correlation function peak plus the time difference between the onset and peak of respective template signal (0.35 ms for SN-template and 3.5 ms for M-template, see Fig 2 and Fig 4 respectively). (3) The magnitude of the cross-correlation function peak served as a measure of the magnitude of the event in arbitrary units, which can be scaled post-hoc to report similar magnitudes as the peak-to-peak value. For N10avRs, the WSA total magnitude (TMag^WSA^) was defined as the sum of all individual event magnitudes quantified by the WSA method in a complex response (e.g. the sum of peaks at arrows in Fig 2 row B). For M5avR, TMag^WSA^ was based on the single EMG event quantified by WSA.

### Event detection and quantification derived by Trained Eye Analysis (*in vivo*)

The identical signals that underwent WSA were also subject to TEA. The subconscious trained eye was used as the TEA method to detect peaks. This was accomplished by an observer who was assigned the task of quantifying all visible peak-to-peak magnitudes for each response, but was unaware that the number of peaks would be quantified from this task. Events that were selected by the observer for peak-to-peak quantification were therefore considered as events detected by TEA. The TEA method chosen to quantify latencies was by manually obtaining the latency of the peak of the first encountered event. The TEA method chosen to quantify response magnitudes was the peak-to-peak value of each event visible to the trained eye, and was measured from the largest local positive peak to its respective largest negative peak. The total magnitude quantified by TEA (TMag^TEA^) for N10avRs was defined as the sum of the peak-to-peak amplitudes of all events from the N10avR (e.g., the sum of peaks at arrows in Fig 2 row A), while TMag^TEA^ for M5avRs were based on the single EMG response.

### WSA algorithm performance (*in vivo* data)

To evaluate the WSA algorithm, event detection, latencies and magnitudes were obtained using the WSA algorithm and compared to values obtained from the identical signals but quantified using TEA. Thus, to examine the ability of the WSA algorithm to detect events, the number of CNAP events that were obtained during magnitude quantification task during TEA was compared to those detected by WSA. In the case of CMAP, where only one event was present, the algorithm was assessed by comparing detection by WSA and TEA following the shortest inter-pulse stimulus intervals, i.e. where responses were absent or minimal. To examine the ability of the WSA algorithm to derive latencies, the latency of the first detected event was measured using WSA, and then subtracted from the latency obtained by TEA of the same event. The absolute difference between latencies derived from the two methods was averaged across all stimuli to derive the mean absolute difference in latency. To examine the ability of the WSA algorithm to quantify magnitudes, TMag^TEA^ was compared with TMag^WSA^ for each stimulus intensity (CNAP) or paired-pulse stimulus (CMAP). Amplitude correction of TMag^WSA^ was performed by multiplying TMag^WSA^ by a constant derived from the ratio of TMag^TEA^ and TMag^WSA^, calculated at the maximum stimulation intensity (1.4 mA, nerve stimulation) or inter-pulse interval (10 ms, muscle stimulation). The same amplitude correction constant was then applied for all other stimulation conditions. All mean values are expressed as mean ± standard error of the mean (SEM).

### WSA algorithm performance (simulated data)

**Fig 5:**
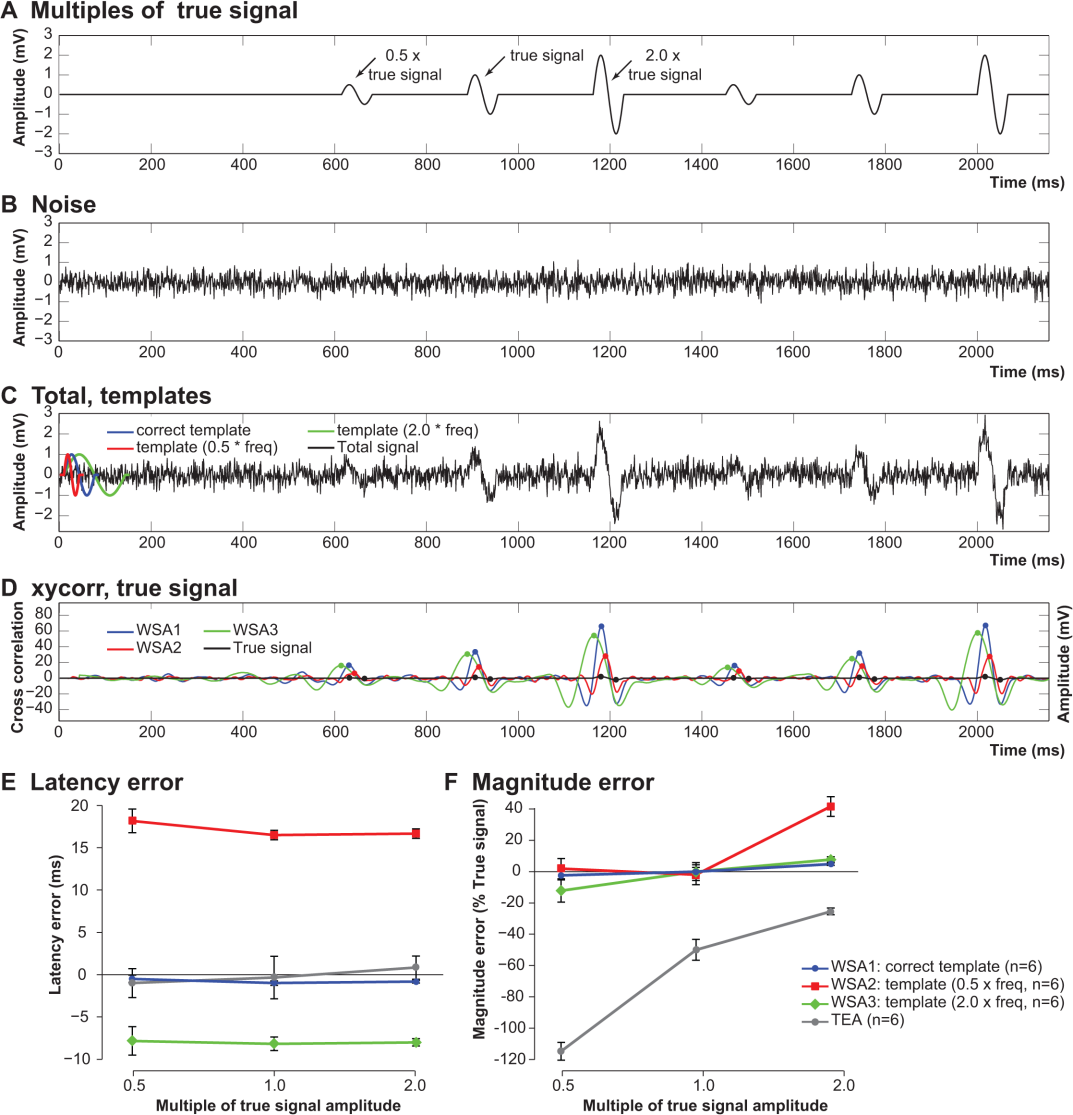
Simulation experiment of WSA performance under conditions of template distortion. Simulated signals generated by adding three multiples (0.5×, 1.0× and 2.0×) of a signal of interest (A, true signal) to noise (B), were used to derive a signal for analysis (C, total). WSA was applied using three templates; the correct one identical to 1.0× the true signal (C, blue), a template with double (C, red) and half (C, green) the frequency content of the correct template. Latency-corrected WSA outputs calculated from the cross-correlation sequence (xycorr) are shown (D) for each template (WSA1, blue, output with correct template; WSA2, red, template with double the correct frequency; WSA3, green, template with half the correct frequency). Overlayed to WSA outputs is the true signal, with the positive and negative peaks indicated (black dots) that reports the true peak-to-peak measurements for comparing the performance of WSA and TEA. Peak detection threshold at >4× the standard deviation of the first 400 ms of each respective WSA output was used to automate peak detection (D, blue, red and green dots indicate peaks for WSA1, WSA2 and WSA3 respectively). The latency (E) and Magnitude (F) errors are shown for WSA performed by each template (following latency and amplitude correction respectively) and TEA for the 6 samples of each signal. The black line indicates zero error; data expressed as mean ± SEM.

To examine the effect of applying WSA with an imperfect template, a simulation experiment was conducted (Fig 5) which used a simple sine wave “true” signal (Fig 5A) with zero-mean normally distributed random noise (Fig 5B) added to generate a “total” signal for analysis (Fig 5C) that represents a typical signal acquired under experimental conditions. Three amplitudes of the true signal were used (0.5×, 1.0× and 2.0×) to examine the performance of WSA under various template conditions. These conditions included the correct template (identical to 1.0× the true signal), and 16 distorted templates ranging from half to double the frequency content of the correct template, which were created by expanding or contracting the original template in time. The latency and magnitudes were automatically detected using 4× the standard deviation of the respective WSA output on a background region of the cross-correlation sequence (i.e. the first 400 ms prior to the first signal of interest). The WSA outputs for each template were the event latency (corrected) and the magnitude (amplitude-corrected to 1.0× true signal: mean peak-to-peak of true signal/mean WSA output). These were then compared to TEA. TEA was performed on a separate figure that only presented the total signal, so that the observer was blind to the location of the true signal. Quantification was performed on 6 samples for each signal magnification (Fig 5 shows examples of two for each signal) and mean and SEM calculated for comparisons. Errors in latency (latency of true signal minus latency determined by WSA or TEA) and magnitude (magnitude of true signal minus magnitude generated by WSA or TEA, expressed as a percent of the magnitude of the true signal) were calculated and expressed as mean ± SEM. See Fig 5A–5D for further details.

## Results

Electrophysiological data with CNAP (n = 6) and CMAP (n = 9) responses was obtained from the stimulation of intact and regenerated nerves of the same animal after 12 weeks of recovery from transection injury of the right sciatic nerve. CNAPs from the intact side had predominantly simple response morphologies, with the occasional animal demonstrating a complex response, while all CNAPs evoked from regenerating nerves always displayed complex response morphologies. The CMAP always demonstrated simple response morphologies, regardless if derived from the intact or regenerating side. There was a relatively poor signal-to-noise ratio associated with responses from regenerated nerves, due to their typically small response magnitudes, compared to background noise levels.

The computer processing time required to quantify the responses by the WSA algorithm was negligible when compared to the human task of selecting the appropriate files to analyse. The WSA algorithm allowed almost instantaneous analysis of peaks and latencies and was able to produce consistent and reproducible results when compared to evaluation by TEA.

### Event detection by WSA

To assess the efficacy of the WSA algorithm to detect CNAPs, the number of events from N10avRs detected by WSA in response to the lowest and highest stimulus intensity was examined, and compared to TEA. At the lowest level of stimulus intensity (0.1 mA) both WSA and TEA detected one or more events form N10avRs of all animals following stimulation of the intact nerve, and the 5/6 animals following stimulation of the injured nerve. There was no significant difference between the mean number of events detected in the N10avRs using WSA (3.0 ± 0.6 events) than by TEA (2.3 ± 0.3) on the intact side, or the regenerating side (2.0 ± 0.4 and 2.5 ± 0.6 respectively). At the highest stimulus intensity (1.4 mA) both methods detected N10avRs in all animal elicited from both nerves. There was a slight, but significant increase (p = 0.04, paired t-test) in the mean of number of events detected by WSA (3.8 ± 0.4) compared to TEA (2.8 ± 0.3) on the left (intact) side, but no significant difference on the right side (5.5 ± 0.2 and 6.0 ± 0.8 respectively).

At shortest inter-pulse interval (0.3 ms) tested, WSA did not detect any M5avRs, while TEA found one response among 9 animals from intact nerves. From the injured side, no events were detected by WSA or TEA at this inter-pulse interval. At the second shortest inter-pulse interval (0.6 ms), both WSA and TEA detected events in 7/9 animals elicited from both intact and injured nerves.

### Response latency quantification by WSA

**Fig 6:**
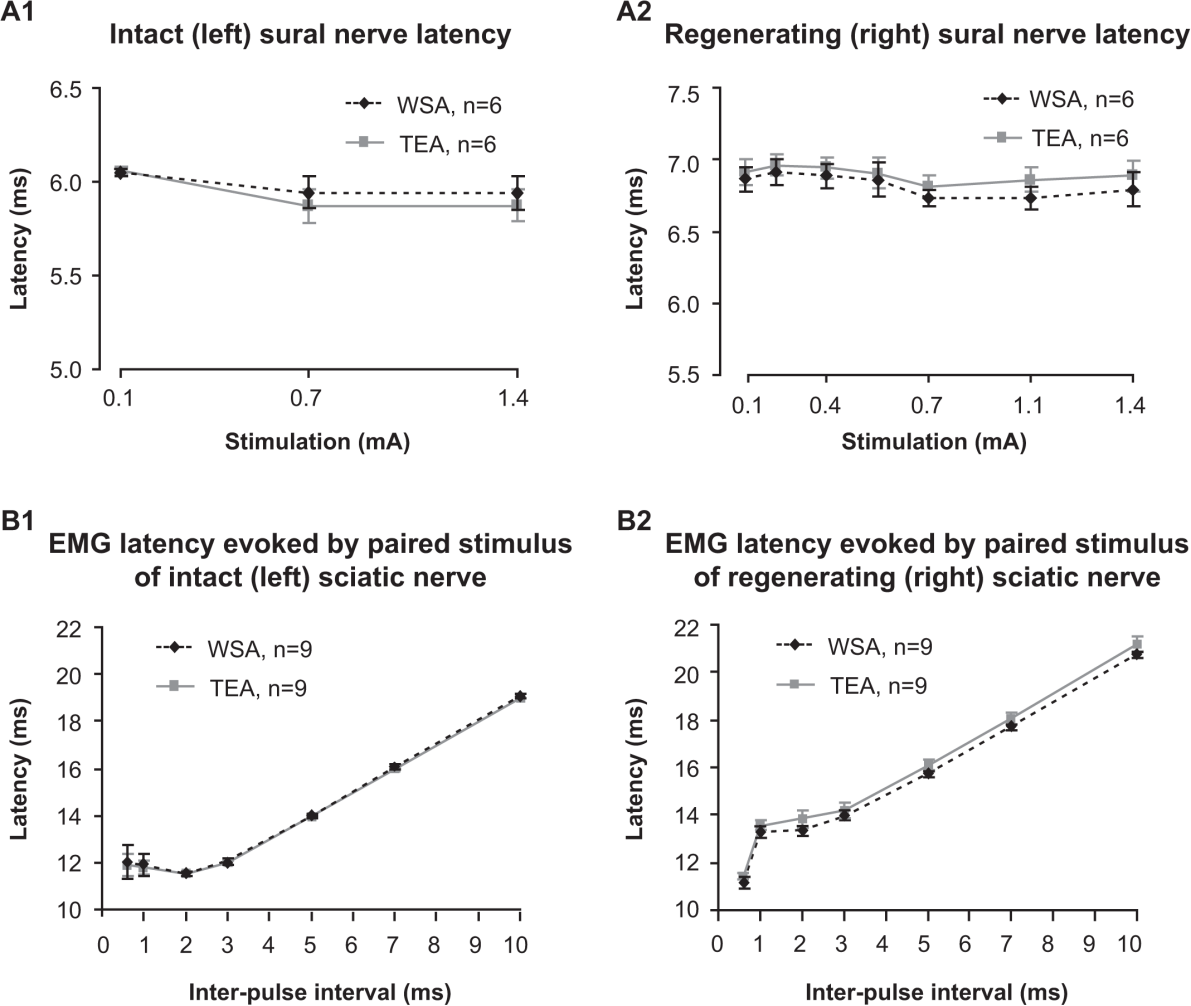
Latency profiles derived by WSA and TEA. The latency to the peak of the first event of N10avRs (A1, intact side; A2, regenerating side) and the latency to the peak of M5avRs (B1, intact side; B2, regenerating side) were measured using the temporal location of best fit between the event and its respective template signals (WSA), and compared with the latency of the peak of each respective event (TEA). There was no significant difference in the latencies derived from WSA and TEA. Inter-pulse interval = time interval between the conditioning and testing pulses (refer to Fig 4). Abbreviations: WSA, waveform similarity analysis; TEA, trained eye analysis.

Latency profiles were produced by quantifying the same CNAP latencies to varying stimulation intensities using WSA and TEA; the intact side received 3 stimulation intensities and the regenerating side received 7 (Fig 6A1 and 6A2). The reduced number of stimulation intensities on the left side was due to limitations in the data set, but this did not impede our ability to evaluate the WSA algorithm and compare it to TEA. There was no significant difference between the two methods used to quantify latency of CNAPs as demonstrated by their latency profiles (Fig 6A1 and 6A2), nor was there a significant difference in the mean absolute difference in latency across all stimuli between the intact (0.08 ± 0.03 ms) and the regenerating sides (0.08 ± 0.01 ms).

Latency profiles for CMAPs were produced by quantifying the CMAP latencies in response to varying inter-pulse intervals. There was no significant difference between the two methods used to determine the latency of CMAPs as shown by their latency profiles (Fig 6B1 and 6B2). The mean absolute difference in latency calculated by the two methods across all paired pulse intervals was 0.09 ± 0.02 ms for intact nerves and 0.35 ± 0.03 ms for regenerating nerves.

### Response magnitude quantification by WSA

**Fig 7:**
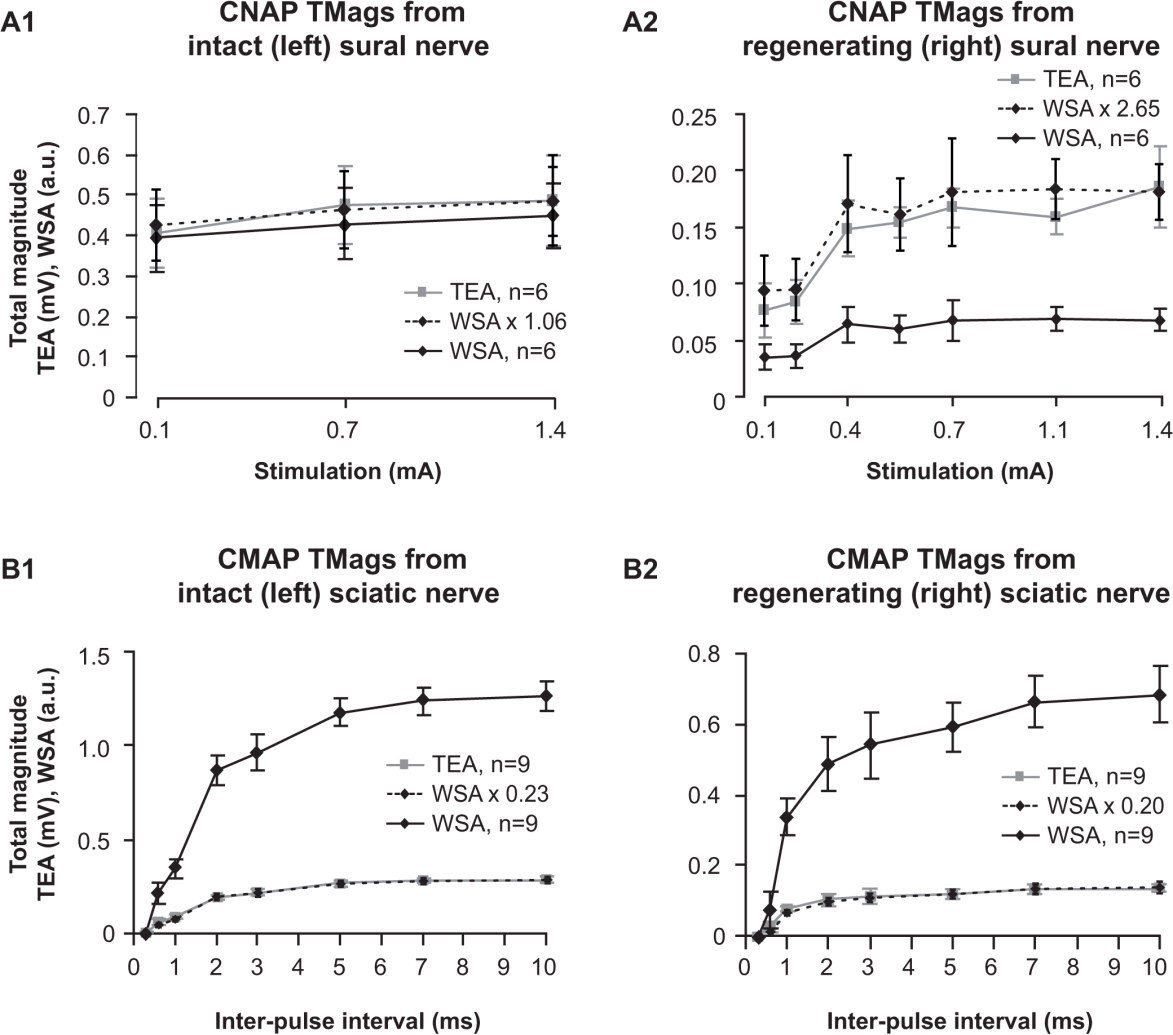
Magnitude profiles derived from WSA and TEA. N10avR total magnitudes (TMags) were calculated by quantifying and summing magnitudes of all events for each response of intact (A1) and regenerating (A2) sural nerves using WSA (black) and TEA (grey). TMags derived from WSA were amplitude-corrected (dashed) by multiplying the TMag^WSA^ by a constant (indicated) derived from the ratio of [mean TMag^TEA^]/[mean TMag^WSA^] (see Materials and Methods) evoked from the strongest stimulus pulse (1.4 mA). Magnitude profiles were also derived from M5avR TMags for intact (B1) and regenerating (B2) nerves. Similarly, TMag^WSA^ of M5avRs were amplitude-corrected (dashed) by multiplying by a constant (indicated) based on the ratio of mean total magnitudes with the greatest inter-pulse interval (10 ms).

Magnitude profiles were generated (as described for response latency quantification above) to enable the comparison of response magnitude quantification by WSA and TEA. TMag^WSA^ demonstrated a consistent and proportional quantification when compared with TMag^TEA^ for both intact (Fig 7A1) and regenerating nerves (Fig 7A2). There was no significant difference between TMag^WSA^ and TMag^TEA^ at any of the stimulus intensities after TMag^WSA^ was amplitude-corrected to TMag^TEA^, indicating that quantification by the WSA method was in agreement with that derived by TEA for both intact and regenerating nerves (Fig 7A1 and 7A2).

The test pulse stimulus induced variations in the CMAP amplitudes and hence the generation of magnitude profiles. The WSA method demonstrated a consistent and proportional quantification when compared to TEA for all paired stimulus intervals, after the amplitude correction of the WSA output was performed (Fig 6B1 and 6B2).

### WSA performance of imperfect templates

To examine the effect of an imperfectly matched template on the output of WSA, a signal with 3 amplitudes was examined using correctly and incorrectly assigned templates. All signals were successfully detected by WSA and TEA during simulation experiments; i.e. there was no effect of distorting the template on signal detection.

The correctly assigned template resulted in small errors in latency which were not significantly different to errors made by TEA for all three signal amplitudes investigated (Fig 5E, 1.0×, blue). There was no significant effect of the signal amplitude on the error for any of the templates or TEA, however, a misspecified template resulted in a consistent error across all three signal amplitudes investigated (Fig 5, 2.0×, red; 0.5×, green). The mean latency error could be predicted as a function of the incorrect frequency of the template as follows:

$$y=\frac{10}{\left(x^{1.55}\right)}-10$$

where *y* = the latency error, and *x* = template frequency mismatch expressed as a multiple of the correct frequency between 0.5× and 2.0× that of the correct template.

Signal amplitude had no effect on the relationship between the latency error and the incorrect template frequency, and the above equation described the experimental observations for all of the three signal amplitudes with large coefficients of determination (0.5× true signal amplitude, R^2^ = 0.984; 1.0×, R^2^ = 0.980; 2.0×, R^2^ = 0.978).

The correctly assigned template, or templates comprising of greater frequencies, resulted in significantly smaller errors compared to TEA at all three signal amplitudes investigated (Fig 5F). At the largest signal amplitude, templates with frequencies of 0.6× or below were outperformed by TEA, however, templates with frequencies of 0.65× (error = -9.4 ± 6.4% true signal amplitude; *p* = 0.0004, Student’s t-test vs TEA) and above outperformed TEA with the mean % error approaching zero (0.7×, error = -1.5 ± 7.6% true signal amplitude; *p* = 0.007).

## Discussion

This study demonstrates that WSA can accurately and reliably detect events and quantify their latencies and magnitudes derived from limited data sets of simple and complex responses with low and high signal-to-noise ratios. Furthermore, the algorithm provides reliable output, even under conditions of significant template distortions. This was true even though the range of distortions explored here was much larger that would commonly occur. Thus, these qualities are useful for evaluating complex noisy responses, such as those produced by regenerating nerves, and therefore serves as a useful tool for investigating recovery from neural injury.

### Event detection

We were able to detect events from signals with relatively large background noise levels using WSA. This is consistent with others who report successful event detection with insensitivity to levels of recording noise and amplitude variability using a similar technique of cross-correlation of two signals for event detection [13]. The greater sensitivity of WSA to signal, and insensitivity to noise, enables event detection with less averaging of evoked responses, and hence less overall stimulation of the nerve. This is of particular relevance for regenerating nerves where there may be concerns regarding the integrity of the nerve and the robustness of its response from prolonged stimulation protocols (personal observations). In our study, we were able to detect events within a complex response with a total of 30 repeated stimulations (3 repeated N10avRs) with a low pass filter of 10 kHz. This trial number is more than 16 times less than previous studies who report that up to 500 evoked responses were necessary in order to distinguish a response from noise with the use of a bandpass filter of 200–6000Hz [2,3]. It must be acknowledged however, that making direct comparisons of this nature is difficult due to the somewhat different experimental conditions.

In the present study we reported a slight increase in the number of detected events evoked by stimulation of the intact (left) sural nerve at the highest stimulation intensity (1.4 mA) using WSA when compared to detection by TEA. This greater number of detected events at the highest stimulation intensity suggests that WSA was more sensitive at event detection than TEA for the detection threshold level used in this study. The greater sensitivity of WSA is likely to be due to the observer failing to notice smaller events alongside a much larger event in the same response in the case of intact nerves. To illustrate this point, consider an example of the event from the intact side illustrated in Fig 2A1 and 2B1 (arrow at approximately 9 ms). This event is slightly larger in magnitude (0.01 a.u.) than the event from the injured side illustrated by the 5^th^ arrow (0.0088 a.u.) at approximately 10 seconds in Fig 2A2 and 2B2. It is conceivable that with the large difference in magnitudes between these events from the intact nerve, an observer could overlook the smaller events such as the 2^nd^ event in Fig 2A1. An observer would however, be less likely to overlook the 5^th^ event in Fig 2A2 due to its similar magnitude with respect to its neighbouring events in that complex response. Furthermore, as the event in Fig 2A1 is in the same order of magnitude as the background noise level, it is well hidden, making its identification almost impossible by TEA. By contrast the WSA threshold of 2SD identifies the event accurately. The greater sensitivity of WSA compared to TEA, in addition to the advantage that it can be automated, makes WSA a more efficient and convenient method to detect events than by the trained eye.

### Resolving response latencies

Despite not being significantly different, the latencies determined by the WSA algorithm were not identical to the latencies derived from the same responses using TEA (see Figs 5F and 6). Latencies determined by TEA depends on a trained observer to identify the position of the perceived local maximum; however the local maximum of an event may not necessarily be the location of the true peak if noise constructively interferes near, or destructively interferes on, the true peak of the signal. The location of maximum similarity, or best fit, with an appropriate template will provide a more accurate measure of an event’s latency, rather than the local maximum/minimum, because it takes into account the entire waveform, and is not solely dependent on a small range confined to the peak of the waveform (Fig 7). Our simulation experiments demonstrated that a template with the incorrect frequency content will induce a fixed latency error for the same signal of different amplitudes, and that the error can be expressed as a function of template frequency distortion (Fig 5E). While we were able to determine an equation to estimate the error in our simulation experiment, it is important to note that the equation is unique to the template itself. Template parameters (e.g. the proportions of positive and negative peaks, the number of peaks in a complex template and/or the composition of frequencies) would alter the relationship between the frequency distortion of the template and the error from a true signal, thus a unique relationship of template frequency distortion and latency error exists for each template. The best performance, equal to that of TEA, was achieved by the correct template, thus knowledge of the signal of interest is essential to maximise the performance of latency estimation by WSA.

### Quantifying response magnitudes

Whilst other studies have used cross-correlation/template comparing techniques for event detection [10–13], to our knowledge cross-correlation has not been utilized to quantify response amplitudes as we have performed here on *in vivo* data. For the WSA algorithm to adequately quantify magnitudes of compound action potentials, it has to consistently quantify the response signal in terms of its relative energy. As mentioned above, the cross-correlation function (*xycorr*) and the peak-to-peak amplitude are both directly related to the energy of the signal. Therefore, to validate WSA as a method for quantifying response magnitudes, it must quantify signals in proportion to the peak-to-peak value (TEA) when presented with the same signal. Here we demonstrated that for all stimuli used in our *in vivo* experiments, WSA derived magnitudes in proportion to those derived by TEA for both CNAPs and CMAPs (Fig 7). There was, however, a lesser degree of “proportionality” evident between magnitudes derived by WSA and TEA when quantifying CNAPs evoked from regenerating nerves (note reduced superposition of doted and grey traces in Fig 7A2), compared to the intact nerves (Fig 7A1) or the CMAPs evoked from either intact or regenerating nerves (Fig 6B1 and 6B2). Signals evoked from the regenerated nerves were of the poorest quality in this study, i.e. the noise levels were of the same order of magnitude as the signal (e.g. Fig 2A2). Quantifying signals of such low quality is laborious and/or unreliable using TEA, as arbitrary decisions (or estimates) must be made by the observer as to what is true signal and noise. Our simulation experiments support this idea by demonstrating that the magnitude error was significantly greater with TEA when quantifying signals of smaller amplitudes (Fig 5F). This is presumably because the relative noise level is greater for small signals. However, the noise level with a mean of zero is of little concern for WSA, as noise in the negative direction will cancel that in the positive direction and thereby not contribute to the overall quantification.

Magnitudes derived from WSA were directly proportional to peak-to-peak amplitudes, therefore if there is a clear and measurable feature in the signal, WSA could be calibrated to accurately estimate peak-to-peak values. In the case of the CMAP, the amplitude correction constant used to scale WSA to the peak-to-peak value was 0.23 on the left side and 0.20 on the right was (see Fig 6), whilst for nerve responses this was greatly different (1.06 and 2.65 respectively). This indicates that WSA cannot be used to directly compare signals with vastly different morphologies, such as for example, the sural nerve responses evoked from intact nerves (e.g. Fig 2A1) that are similar to the template signal, with responses evoked from regenerating nerves which have comparatively less-similar features to the template signal (e.g. Fig 2A2). Nevertheless, WSA successfully quantified responses within the same group, i.e., there was consistent quantification of nerve responses within the injured group, as indicated by the proportionality between quantification using TEA and WSA. Our simulation experiments indicate that consistent magnitude quantification will occur even if the evaluating template is significantly distorted. Thus, even with an imperfect template signal, WSA can consistently and reliably quantify signals of similar morphology.

### Template shape

The most significant limitation of WSA is that quantification is dependent on the shape of the two signals being cross-correlated. Thus, the choice of the template signal is crucial when quantifying latencies and magnitudes, as the template must have some resemblance to the overall shape of the signal of interest. In this study, we used a template signal derived from an average of signals from the intact side from all animals. Averaging responses from multiple animals provides a robust approximation of the true signal by reducing the noise while conserving the essential frequencies that comprise the true signal. Simulation experiments indicated that the template frequency could be comfortably distorted with the range of 70–200% without having a significant impact on event detection or magnitude quantification, however, the correct template is required for accurate estimation of the latency. Nevertheless, the latency error is a fixed value and therefore may not be important in some experimental designs, particularly where only a relative change in latency is needed.

## Conclusion

WSA robustly detects and quantifies events in terms of magnitudes and latencies and performs consistently when compared to the trained eye. It is relatively insensitive to noise, is automatic and thereby enables objective measurements that can be applied to signals of similar morphology with a forgiving degree of dissimilarity to the template waveform. Magnitude is quantified in arbitrary units of energy, and thus can be calibrated to an equivalent peak-to-peak measurement. WSA is therefore ideally suited to detecting events under conditions of considerable noise levels that would otherwise be difficult or impossible with the trained eye, such as signals derived from regenerating or developing excitable tissues.
